# A Rat Model of Clinically Relevant Extracorporeal Circulation Develops Early Organ Dysfunctions

**DOI:** 10.3390/ijms24087338

**Published:** 2023-04-16

**Authors:** Antoine Persello, Fouzia Souab, Thomas Dupas, Virginie Aillerie, Edith Bigot, Manon Denis, Angélique Erraud, Thomas Pelé, Angélique Blangy-Letheule, Pierre Miniou, Philippe Guedat, Michel De Waard, Emmanuelle Abgueguen, Bertrand Rozec, Benjamin Lauzier

**Affiliations:** 1 Nantes Université, CHU Nantes, CNRS, INSERM, l’institut du thorax, F-44000 Nantes, France; 2 InFlectis BioScience, 44200 Nantes, France; 3 Department of Biochemistry, CHU de Nantes, 44800 Nantes, France

**Keywords:** extracorporeal circulation, rat model, cardiac surgery, patients cohort, transcriptomics

## Abstract

In clinical practice, extracorporeal circulation (ECC) is associated with coagulopathy and inflammation, eventually leading to organ injuries without preventive systemic pharmacological treatment. Relevant models are needed to reproduce the pathophysiology observed in humans and preclinical tests. Rodent models are less expensive than large models but require adaptations and validated comparisons to clinics. This study aimed to develop a rat ECC model and to establish its clinical relevance. One hour of veno-arterial ECC or a sham procedure were achieved on mechanically ventilated rats after cannulations with a mean arterial pressure objective > 60 mmHg. Five hours post-surgery, the rats’ behavior, plasmatic/blood biomarkers, and hemodynamics were measured. Blood biomarkers and transcriptomic changes were compared in 41 patients undergoing on-pump cardiac surgery. Five hours post-ECC, the rats presented hypotension, hyperlactatemia, and behavioral alterations. The same patterns of marker measurements (Lactate dehydrogenase, Creatinine kinase, ASAT, ALAT, and Troponin T) were observed in both rats and human patients. Transcriptome analyses showed similarity in both humans and rats in the biological processes involved in the ECC response. This new ECC rat model seems to resemble both ECC clinical procedures and the associated pathophysiology, but with early organ injury corresponding to a severe phenotype. Although the mechanisms at stake in the post-ECC pathophysiology of rats or humans need to be described, this new rat model appears to be a relevant and costless preclinical model of human ECC.

## 1. Introduction

Extracorporeal circulation (ECC) is an essential procedure in both critical care and cardiothoracic surgery to support or replace the heart and/or pulmonary function. ECC includes three procedures: extracorporeal membrane oxygenation (ECMO), extracorporeal life support (ECLS), or cardiopulmonary bypass (CPB) [1]. Despite the numerous benefits, ECC can also result in complex adverse events [2]. Complications include acute kidney injury, respiratory dysfunction, cardiac dysfunction, neurological issues, and multi-organ dysfunctions (MODs). Blood contact with artificial surfaces and air, surgical stress, long on-pump times, multiple procedures, and difficulties achieving an appropriate tissue blood supply and oxygenation are associated with a strong inflammatory response and poor clinical outcomes. The mechanisms involved include microcirculatory and metabolic disorders, ischemia reperfusion, activation of the complement, kinin, fibrin pathways and inflammation [3,4], and bacterial endotoxin translocation from the digestive system. Unfortunately, MODs occur relatively frequently to varying degrees following cardiac surgery with CPB or ECMO/ECLS [4]. Various research aims to develop new methods and compounds to address these issues and improve patient outcomes. Study methods implicate the use of study models, such as bioinformatic models, animal models, and human clinical trials. The latter remains the most reliable but is also the most limited. To date, animal models mimicking the pathophysiology of ECC is the most relevant approach, especially swine models, which remain the most relevant to humans in terms of anatomical structures and physiology, but these are expensive and resource intensive. On the other hand, rodent models are less expensive in comparison, allowing an increased range of testing that is still comparable to human physiology, particularly for cardiovascular function. However, there many are deviations from human physiology that need to be understood, such as higher metabolic rates, slightly different cardiac electric activity and structure, and a higher heart rate [5,6,7]. The ECC rat models described thus far have made it possible, for example, to study in a reproducible way the weaning of ECC and the impact of anti-coagulants, hypothermia, and cardioplegia, without recovery in most cases [8,9,10]. However, the main difficulty associated with these models lies in the miniaturization of the device and protocols [11]. Similarly, the difficulty of accessing different anatomical structures can largely complicate several protocols, such as thoracotomy surgery and aortic clamping if the animal’s recovery is a necessary step [12]. This complexity is the first difference with procedures used in clinical settings. The models are commonly generated by peripheral vascular canula placement (femo-femoral or caudo-femoral) excluding central canula placement (right atrium and aortic arch). Hence, extensive comparison to human responses with modern tools is rare and, to date, no study has made the comparison at both the macroscopic and molecular levels. The first aim of this study was thus to develop a reproducible rat model of ECC developing MODs after recovery, closer to human CPB procedures in terms of the surgical approach, pharmacology, aims, and monitoring. The second aim was to investigate how reliable this model is regarding the ECC effects compared to humans in the context of clinical practice by a direct comparison of in vivo monitoring and plasmatic biochemistry. Standard monitoring of hemodynamic and plasmatic markers measures was completed with an analysis of the PBMC’s transcriptome by the DGESeq method and biological processes analysis in both patients and rat models. This approach strengthens the description of a rat model of ECC and clarifies what can be compared between the patients and models. This finding could help refine tests of therapeutic approaches or devices in the context of ECC.

## 2. Results

### 2.1. Hemodynamic, Lactate, and Hemoglobinemia Measures in Rats

**Figure 1:**
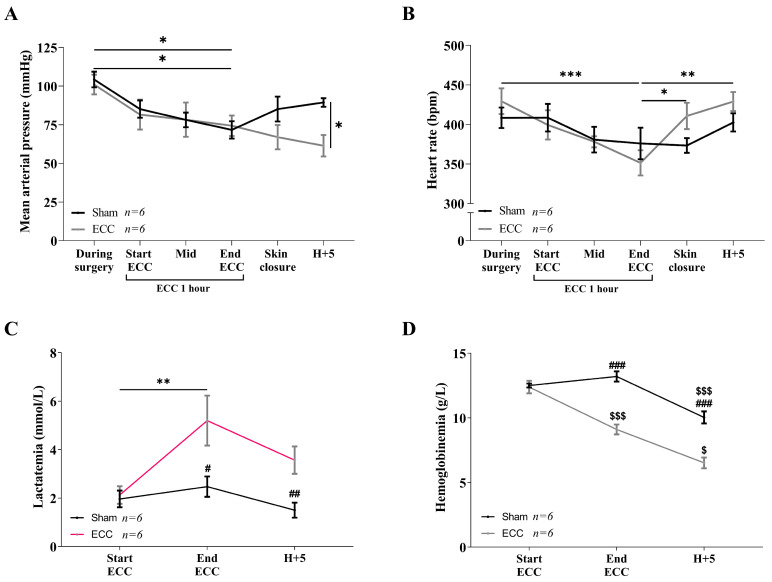
Hemodynamic monitoring, lactatemia, and hemoglobinemia measures in rats over time. The sham group (*n* = 6) is represented in black and the ECC group (*n* = 6) in grey. The mean arterial pressure (mmHg) (**A**) and heart rate (bpm) (**B**) were monitored during the procedure. Each point (during surgery, start of ECC, mid-ECC, ECC end, skin closure, and H+5) is a mean of 10 min of recording. Lactatemia (mmol/L) (**C**) and hemoglobinemia (g/L) (**D**) were measured from point-of-care tests from whole arterial blood at the start of ECC, at the ECC end, and 5 h postoperatively (H+5). Statistical analyses were performed by a paired two-way ANOVA test with Sidak’s multiple comparisons test. * *p* < 0.05, ** *p* < 0.01, *** *p* < 0.001, # *p* < 0.05 vs. ECC, ## *p* < 0.01 vs. ECC, ### *p* < 0.001 vs. ECC, $ *p* < 0.05 vs. ECC start, $$$ *p* < 0.001 vs. ECC start.

The mean arterial blood pressure (MAP) and heart rate (HR) were monitored to validate that ECC was sufficient to achieve the MAP objectives (Figure 1A,B) and to objectify perioperative hemodynamic instability. No differences were found between the sham and ECC groups during surgery or until the end of the procedure. The ECC group presented significant hypotension at H+5 (ECC 61.38 ± 6.95 mmHg vs. Sham 89.39 ± 2.73 mmHg, *p* < 0.05). During the ECC period, the ECC groups showed an increase in blood lactates (Figure 1C) over time (ECC start: 2.13 ± 0.33 mmol/L vs. ECC end: 5.20 ± 0.95, *p* < 0.05). The lactate levels in the sham group remained significantly lower than those in the ECC group at the conclusion of ECC and H+5 (*p* < 0.05). Hemodilution was observed (Figure 1D) in the ECC group from the termination of ECC (*p* < 0.01), which worsened at H+5 (*p* < 0.001), resulting in a significant difference in hemoglobinemia in the sham group at the end of ECC (*p* < 0.01) and at H+5 (*p* < 0.01).

### 2.2. Blood Gases, Electrolytes Measure in Whole Blood of Rats

The blood gases, blood cell counts, and electrolytes were measured before the start of ECC, at the end of ECC, and at H+5 to validate the preservation of acid–base, ventilatory, and blood parameters in the model (Table 1). As expected, the blood gases showed values in the normal range and no significant changes over time or between the groups. Concerning the electrolytes, a slight but significant hyperkaliemia was observed in the ECC group 5 h post-operatively (sham: 4.13 ± 0.13 mmol/L; ECC: 5.03 ± 0.21; *p* < 0.05). A decrease in calcemia was also measured at H+5 (1.10 ± 0.04 mmol/L vs. ECC end: 1.40 ± 0.02 mmol/L, *p* < 0.001) with no significant differences when compared to the ECC start. Five hours post-operatively, calcemia in the ECC group was lower than in the sham group (Sham: 1.24 ± 0.03; ECC: 1.10 ± 0.04 mmol/L; *p* < 0.001).

### 2.3. Patient Demography

Forty-one patients were included, with ¼ of the patients being women, and the mean age was 66 years (Table 2). The patients mainly underwent elective cardiac surgery with a single- or two-valve procedure. All the patients received inotropic or vasopressor support during and after surgery. The mean ECC duration was 126.9 min, the mean ventilation duration was 13 h, and only one patient developed a pulmonary infection, diagnosed in accordance with the criteria for care-associated pneumonia defined by the French societies of critical care (SFAR/SRLF) [13].

### 2.4. Plasmatic Markers in Humans and Rats

**Figure 2:**
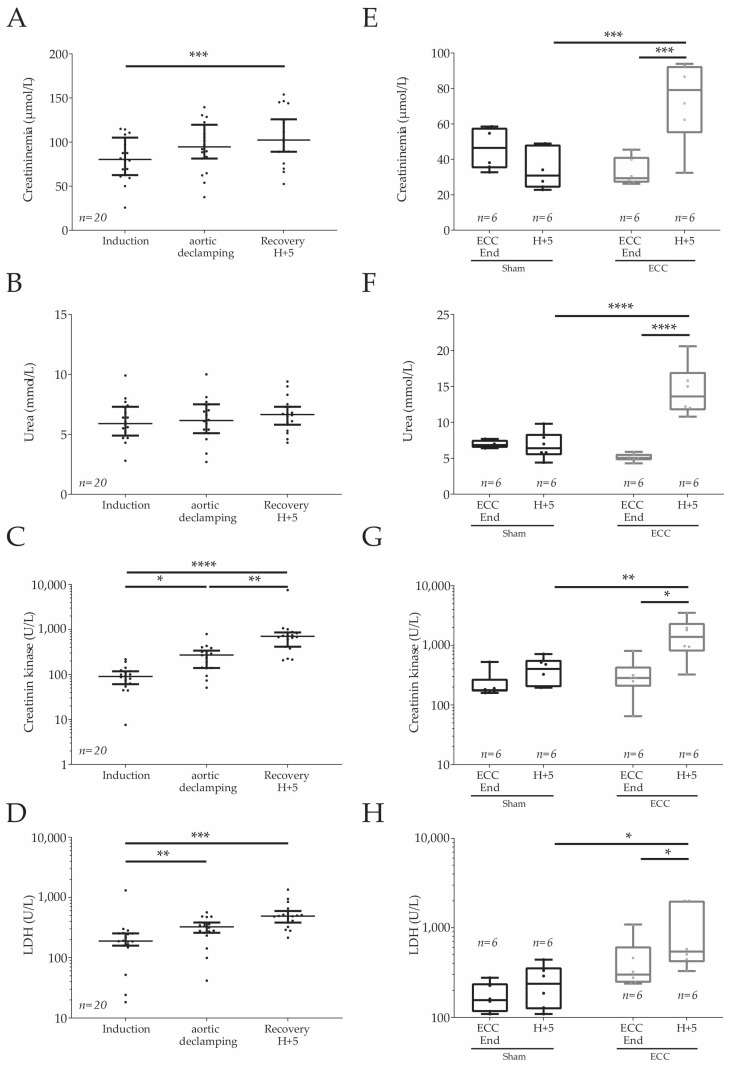
Plasmatic measure of renal markers and stress markers from human and rat arterial blood. The plasma samples were recovered from human blood (**A**–**D**) at the induction of anesthesia, the aortic declamping, and 5 h post-declamping during recovery and from rats (**E**–**H**) at the start of ECC, at the ECC end, and 5 h postoperatively (H+5). The renal markers measured were creatinemia (µmol/L, panels (**A**,**E**)) and urea (mmol/L; panels (**B**,**F**)). Stress was evaluated with the measure of lactate dehydrogenase or LDH (U/L; panels (**C**,**G**)) and creatine kinase (U/L, panels (**D**,**H**)). Statistical analyses were made with paired tests, one-way ANOVA for humans (**A**–**D**) with Dunn’s post-hoc test, and a two-way ANOVA test with Sidak’s multiple comparisons test for rats. * *p* < 0.05, ** *p* < 0.01, *** *p* < 0.001, **** *p* < 0.0001.

Among the plasma biomarkers in patients, the renal biomarkers were measured and showed a significant increase in creatinine (Figure 2A) from induction to 5 h post-operatively (ECC Start: 83.97 ± 5.39; H+5: 105.86 ± 6.58 mmol/L; *p* < 0.001), while there was no modification of urea plasma concentrations (Figure 2B). Stresses, mostly hepatic and muscular, were observed by measuring the plasma markers lactate dehydrogenase (LDH, Figure 2C) and creatinine kinase (CK, Figure 2D). The patients showed an increase in LDH over time (induction: 245.7 ± 65.47; aortic declamping: 316.3 ± 32.2; H+5: 541.2 ± 64.3 U/L), which was significantly different at the aortic declamping (*p* < 0.01) and 5 h post-declamping (*p* < 0.001) in comparison to the induction time. In addition, they presented an expected increase in CK over time (induction: 95.23 ± 12.22; aortic declamping: 271.90 ± 41.10; H+5: 1024.0 ± 386.5 U/L), which was significant at the aortic declamping (*p* < 0.05) and 5 h post declamping (*p* < 0.01) when compared to induction time, but also between declamping and 5 h later (*p* < 0.001).

As for the human parameters, the rats in the ECC groups (Figure 2E–H) presented an increase in creatinine (ECC end: 32.89 ± 3.21; H+5: 73.15 ± 9.58 U/L, *p* < 0.05), LDH (ECC end: 438.45 ± 133.70; H+5: 976.55 ± 326.17 U/L, *p* < 0.05), and creatinine kinase (ECC end: 333.45 ± 101.18; H+5: 1586.13 ± 457.30 U/L, *p* < 0.05). However, rats that underwent the ECC procedure presented a significant increase in plasmatic urea (ECC end: 5.18 ± 0.22, H+5: 14.4 ± 1.46 mmol/L, *p* < 0.0001) that was not found among the human patients. In the ECC group, the creatinine, urea, LDH, and creatinine kinase levels were also higher at H+5.

### 2.5. DEGSeq Analyses

**Figure 3:**
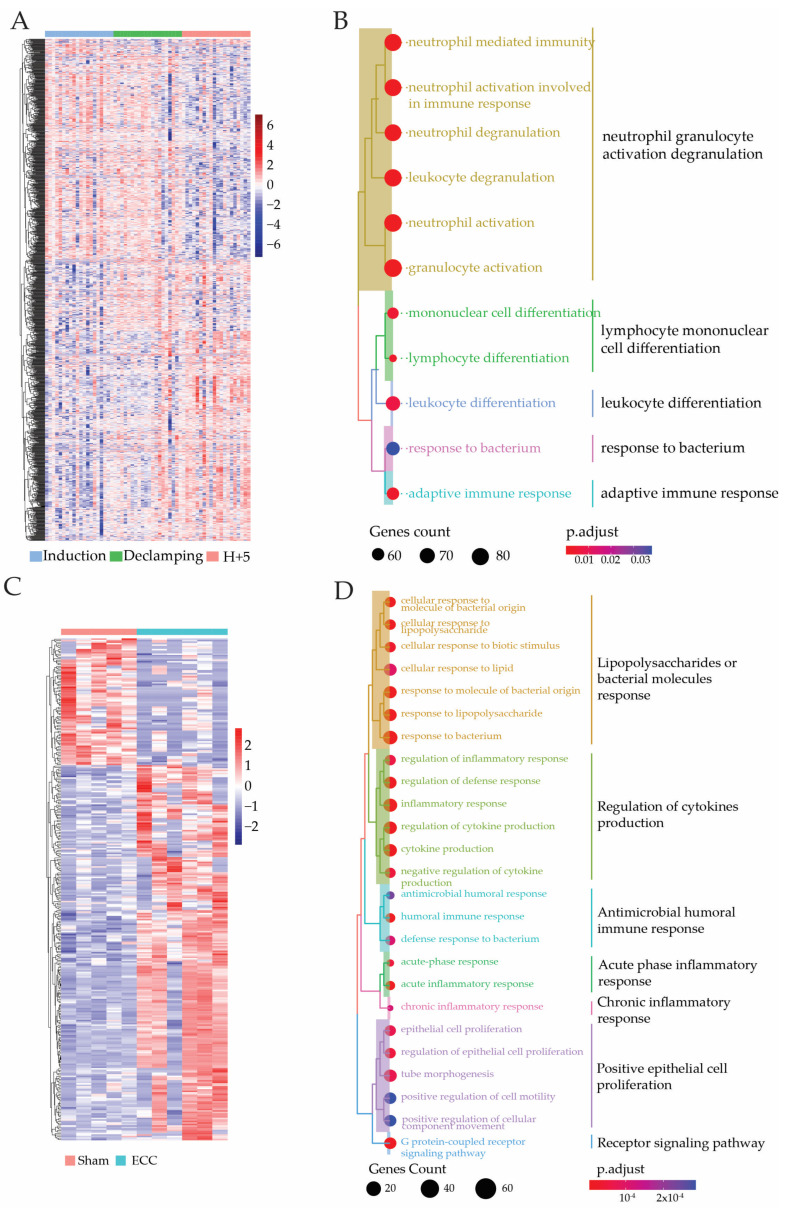
Pathway enrichment analysis from transcriptome analyses from rats’ and patients’ PBMC. The transcriptome analyses were made from PBMC from rats that underwent the ECC procedure (ECC group) or not (sham group). Differentially expressed genes were selected by their fold change of expression (log2Ratio(ECC/Sham) > 2 or log2Ratio(ECC/Sham) < 2), as seen in the heatmap (**A**). The biological process enrichment has been processed on the selected genes and represented in the function of the GeneRatio and count (**B**). The transcriptome analyses were achieved on human PBMC collected before, during, and after the ECC procedure in cardiac surgery. The top 8% of differentially expressed genes were selected by their rank given by the Hotelling T^2^ score calculated with the time course analysis. The selected genes were clustered in the heatmap (**C**). The biological process enrichment has been processed on the selected genes and is represented in the function of the GeneRatio and count (**D**).

The analyses were performed by DGESeq on PBMC isolated at the ECC end time on a new set of operated rats undergoing ECC or sham procedures. After the analyses, 4/15 samples presented an insufficient number of reads or detected genes and had to be excluded from the analyses. A total of 333 differentially expressed genes (Supplemental Table S1) between the sham and ECC groups were found with a transcript expression log2Ratio (ECC/Sham) > 2 or log2Ratio (ECC/Sham) < 2 (Figure 3C). The heatmap presented in Figure 3C represents 333 differentially expressed genes in PBMC. This allowed the representation of two main clusters of genes that were either overexpressed in the sham group or in the ECC group.

Gene ontology enrichment of these sets of genes have been made. As many biological process ontologies shared the same gene set under slightly different names (Supplemental Table S1), they were grouped by semantic similarities. Ontology research showed, without surprise, that the biological processes that were mainly impacted by the ECC procedures concerned the acute inflammation process and the response to bacterial components (Figure 3B).

Numerous genes involved in the inflammatory process were overexpressed in the ECC group, such as the interleukin-1 beta (*IL*-*1β*), the transferrin (*Tf*), interleukin-1 alpha (IL-1α), the nitric oxide synthase 2 (*NOS2*), the tumor necrosis factor (*TNF*), and multiple other cytokines, chemokines, and cytokine receptors (*Il1r2*, *IL1r1*), such as *S100A8* and *S100A9*, which are involved in the modulation of inflammation, leukocytes recruitment, and cytokine secretion. The ontologies of bacterial or infection responses are related to the inflammatory process and comprise genes already involved in the inflammatory process evoked earlier, as most of the genes involved in these processes are already known, described, and annotated in inflammatory disease (i.e., sepsis, shock) or in ECC or surgery-induced inflammation.

Time course analyses were performed to consider the effect of overtime on gene expression in PBMC isolated from the whole arterial blood of each patient. Six samples did not pass the quality control and were excluded from the dataset. This resulted in the exclusion of 6/26 patients for the analyses. The top 8% of ranked genes based on Hotelling’s T^2^ score were retained for the analyses (Figure 3C), which totals 1077 genes of the 13,466 detected (Supplemental Table S1). After the gene ontology enrichment analysis, a semantic similarity analysis was performed to regroup highly similar process groups.

The gene ontology enrichment (Figure 3D) of this set of genes did not show, as it could be expected, a clear increase in inflammatory response as it has been observed in rats. Surprisingly, no significant modification of the well-recognized inflammatory marker gene expression was observed in PBMCs from patients. However, this analysis revealed that the biological processes mainly impacted by ECC procedures in humans are involved in the acute response to bacterial components, particularly neutrophil and leukocyte recruitment and activation and neutrophil degranulation. These last processes re-group genes coding for a cluster of differentiation (for example, *CD63*), inflammatory markers (*S100A9* and *S100A8*, which are also overexpressed in rat models), and small GTPases (*RAC1*).

Even if the model and patient did not share many overexpressed genes in this analysis, the processes involved seem to be highly related. However, these differences in gene expression were not surprising since the animal model used young male adult healthy animals at one time and the patients presented underlying conditions, potentially with an ongoing inflammatory disorder.

## 3. Discussion

There is a need to develop animal models that reproduce ECC, particularly ECLS and CPB, to mimic and study their complications. Although large animals have the advantage of being closer to humans in terms of physiology, they are also associated with higher experimental costs and technical difficulties. Small animal models are also similar to humans for a fair comparison of numerous parameters (e.g., blood pressure and its regulation, acid–base regulation, basic metabolism, and inflammatory response). This makes them suitable for studying organ and tissue insults, changes in physiological parameters (e.g., embolism, hypoperfusion, MODs, temperature disturbances), and for increasing the rate of pharmacological tests. Nevertheless, their size makes adaptation to surgical procedures challenging.

In this study, we present a new rat model of ECC with recovery relevant to the study of early organ dysfunction and hemodynamic instability following ECC. A direct comparison between humans and rats at the macroscopic and molecular levels has also been performed. The novelty of this model lies in the combination of recovery after the procedure, central cannula placements (aortic and caval), and a comparable anesthesia protocol. Central cannula placements allowed the limitation of Harlequin syndrome during the procedure [14], making the protocol relevant to CPB, not just in humans, but also in large animal models. In contrast, other models have predominantly used caudal and/or femoral canulations in rodents, which makes the procedure closer to ECMOs [11,15,16]. A few similar models of CPB [11,17] and new models of cardiac arrest [9,12,18] have been described in the literature using this approach without attempting to recover or using anesthetic protocols that are similar to those in the clinic. The model used by Peterss et al. and described by Günzinger et al. is of particular interest because their thoracic surgical approach for aortic canulation that is closer to the clinical procedure [12,18]. This model allows researchers to perform a relevant cardioplegic arrest with CPB and a post-CPB follow-up under anesthesia. As with their model, our circuit allows passive venous drainage that could be adapted with a suction system, as seen in clinical practice; however, the circuit components’ materials and priming solutions are different in the two models. In addition, both models allow partial or total replacement of cardiac output and gas exchange. The use of an occlusive pump with a limited number of rollers (three in the model) allows a decrease in pressure and crushing of the tubing, limiting the risk of hemolysis.

As described recently, limiting the circuit volume also prevented the appearance of an excessive and early inflammatory response, thanks to a limited contact area between blood and the circuit components [19]. This strategy allowed us to achieve normo-volemic hemodilution with a hematocrit value of >20% for ECC maintenance, which is consistent with clinical observations and contributes to limited mortality and potential acute renal failure [20,21,22]. Continuous monitoring of the hemodynamics indicated that the mean arterial blood pressure was maintained above the desired target of 65 mm Hg in our model. In contrast, common cardiopulmonary bypass procedures aim to reach the cardiac index or output with the pump and set pressures with vasopressor use. To date, no consensus has been reached regarding targeting specific mean arterial pressure during ECC procedures due to a lack of existing evidence [23]. Nevertheless, it is admitted in non-hypothermic conditions that blood pressure should be higher than 50 mmHg to allow minimal and efficient blood supply to organs [24]. Also, the target should be adjusted for each patient according to several parameters, as proposed in a study by Hori and collaborators [25]. In the latter, blood pressure monitoring following ECC weaning indicates that ECC, and probably vasopressor use, are responsible for a hemodynamic instability and hypotension after weaning [26]. Hemodynamic instability could also contribute to a persistent hyperlactatemia. In patients, such increase in blood lactates is strongly correlated to poor oxygen delivery to tissue and organs, resulting in increased morbimortality [27,28]. In our model, with an FiO_2_ of 1.0, and despite the hemodilution that worsens the oxygen transport, a high PaO_2_ and 100% oxygen saturation was observed.

The increase in creatinemia and uremia are also consistent with an early renal injury (AKI), which is confirmed by anuria. These alterations are similarly reported in humans, albeit to a lesser extent, potentially indicating that our rat model presents a more severe kidney dysfunction [29]. Indeed, the KDIGO score for the assessment of kidney failure confirmed that most human patients do not present kidney failure in this study, which is consistent with the literature [30,31]. Acute kidney failure or AKI during and after CPB is currently a major research challenge [30,32,33] for both biomarker and therapeutic research. AKIs are the consequence of common adverse events, including hemodilution, hemolysis, inflammation [34], neuro–hormonal changes, and vasopressor use and misuse during CPB in cardiac surgeries. However, the greater incidence of AKI in the rat model than in clinical practice can be explained by several factors. The first and most important reason is that the urine output of rats was absent from the anesthesia induction to the anesthesia weaning. At the time of the model development, no suprapubic catheters were placed to relieve the kidneys and Foley catheters cannot be placed due to anatomical particularities of male rats. If this was not a problem for sham rats, the ECC rats were exposed to a hemodilution, and a possible fluid overload was in favor of kidney congestion, hypoperfusion, decreased renal perfusion, and glomerular filtration [35,36]. The second reason for the development of early AKI in our model could be related to our choice of phenylephrine as a vasoconstrictor, which could be associated with more AKIs, as suggested in a study of Magruder et al. [37]. Nevertheless, in this latest work, a goal-directed perfusion, with attentive monitoring and a bundle of care, could be associated with a reduced incidence of AKI [37]. Unfortunately, although we tried to develop the closest model of ECC in rats in our study, this goal-directed perfusion is not yet available in small animals (partly related to the absence of miniaturized devices to monitor various ECC physiological values), but could be an interesting improvement to make.

The plasma levels of alanine and aspartate aminotransferase, creatine kinase, and lactate dehydrogenase were comparable in the rat model we developed and in humans. Unsurprisingly, the increase in troponin T concentration, a marker of cardiac stress, was much higher in humans than in rats. This can easily be explained by the fact that no surgical intervention or cardioplegia (cold or warm) has been performed on rat hearts. As the model setup allowed us to mimic both the procedure and response to ECC, we wanted to validate that the signaling pathways involved in the response to ECC in rats were also comparable to those observed in human patients. For this purpose, we performed a transcriptomic study on rats’ and patients’ blood PBMC isolated in similar conditions. In both cases, this analysis showed an important increase in the expression of genes involved in the regulatory pathways of inflammation and the response to pathogens. The most represented biological processes are the regulation of the activation and degranulation of neutrophils, leukocytes, and granulocytes. The demonstration of an effect on inflammation during and after bypass surgery is consistent with the literature, in which characterization of the renal transcriptome by microarray analysis of a bypass surgery rat model indeed shows an important involvement of these pathways [38]. Finally, this study allows us to demonstrate no major differences between the human and the rat model.

### Limitations

The first limitation lies in the difficulty of a thoracic surgery approach in rats that results in the inability to achieve aortic clamping and a cardioplegia protocol with the perspective to wake the animals up after surgery. This makes it difficult to completely mimic CPB procedures in small animals. Additionally, at the end of ECC, no hemoconcentration was adapted to the rat model, making it more susceptible to organ failure. This risk is also potentially increased by hyperoxygenation, although some studies suggest that hyperoxygenation promotes oxidative stress [39] and inflammation in rats [16]. In humans, hyperoxic conditions are often limited to avoid the overproduction of reactive oxygen species and inflammation. All these limitations increase the risk of organ failure.

## 4. Materials and Methods

### 4.1. Clinical Study

#### 4.1.1. Ethics Statement and Inclusions

The samples used herein came from the IBIS collection of human biological samples, located in BRC of the Nantes University Hospital. The subjects’ written consent was collected a day before the intervention. The biological samples were integrated into the human collections of the immunology research program declared in 5 September 2011 under the n° DC-2011-1399 and in the following amending declarations (DC-2012-1555; DC-2013-1832; DC2014-2206 and DC-2017-2987 currently pending) at the Ministry of Research. It underwent a favorable decision from the CPP Ouest IV on 7 April 2015. From December 2019 to March 2021, 41 adult patients (>18 yrs) undergoing elective cardiac surgery with ECC (>60 min) were included (Supplemental Figure S1). All the patients received general anesthesia and postoperative management according to standard clinical practice. The surgery involved at least one valve replacement or at least the ascending aorta (e.g., Bentall procedures). The blood samples had to be processed within two hours to be included.

#### 4.1.2. Clinical Data, Blood Sampling, and Processing

EDTA-anticoagulated blood samples were drawn at anesthesia induction, at aortic declamping, and 5 h post-declamping. One mL of blood plasma was stored and the PBMC were isolated by centrifugation, frozen at −80 °C, and stored. Blood samples of 16 mL were collected on EDTA at three times, respectively: at anesthesia induction, at aortic declamping, and 5 h post-declamping. One mL of total blood was first collected, and 15 mL was then centrifuged at 1700 g for 10 min at 20 °C. The plasma was aliquoted. The blood pellet was resuspended with sterile DPBS (Dulbecco phosphate sterile buffer, Gibco, ThermoFischer, Illkirch, France) to 30 mL and was gently homogenized and carefully placed on a 20 mL layer of sterile Ficoll (FicollPaque Premium, GE Healthcare, Buc, France) in a 50 mL sterile Falcon tube and centrifuged for 35 min at 400 g, 20 °C. The peripheral blood mononuclear cell (PBMC) layer was then collected and washed in 50 mL of sterile DPBS. Finally, the cells were collected and suspended in 500 µL of RLTplus solution for RNA extraction according to the manufacturer instructions. All the samples were stored at −80 °C.

### 4.2. Rat Model Study

#### 4.2.1. Ethical Statement and Animal Care

All the procedures were performed in compliance with the regional ethic committee (protocol 19562, CEEA—Pays de la Loire, France) according to Directive 2010/63/EU of the European Union. The reporting is in accordance with current ARRIVE guidelines. The protocols were implemented on 12–14 weeks old Wistar rats (400 ± 50 g; Charles River Laboratory, les Oncins, France). Our animal care facility received the rats 15 days before surgery to adapt to the new environment. The animals were held in groups of 2–3 at a temperature of 22 ± 2 °C with a humidity level of 55 ± 10%), on a 12 h light/dark cycle and ad libitum access to water and food. Only males were used for this study to have easy access to a vein (penile vein) for preoperative injections.

#### 4.2.2. ECC in Rats

Circuit preparation: The ECC circuit (Figure 1) consists of an open venous reservoir (modified 10 mL syringe; Terumo, Guyancourt, France) that flows to Tygon^®^ tubing (inner diameter, ID, 3.2 mm, 25 cm long; MasterFlex, ColePalmer, Villepinte, France) through a peristaltic pump (3 rollers; Watson-Marlow, Falmouth, UK). This tube supplied a membrane oxygenator (Micro-1-rat oxygenator; Kewei Rising, Shenzhen, China) with blood (3.7 mL of dead volume). The oxygenated blood passed through a 3-way valve allowing sampling for blood gas measurements (BG) and supplied the arterial cannula (23 G microperfuser mounted with a PE-50 catheter; Artsana, Saint Denis, France). The venous reservoir was equipped with an injection port allowing the injection of treatments and was filled with a balanced solution of Ringer-lactate (B. Braun, Saint Cloud, France). A Y-connector (ID = 1.6 mm) allowed the arrival of venous blood by gravity from the vena cava (multi-perforated cannula; NutriSafe 2; 6 Fr; Vygon, Écouen, France) and the infusion of anesthetic during the CPB. The injection port was mounted on the venous reservoir to allow per-CPB propofol/fentanyl infusion, treatment, or filling administration. Finally, a shunt was set up between the venous reservoir and the arterial line to allow bubble removing from the circuit (Figure 1). Prior to the surgery, the circuit was purged, then primed with a total of 7 mL of a balanced Ringer-lactate solution (B. Braun, Saint Cloud, France).

**Figure 4:**
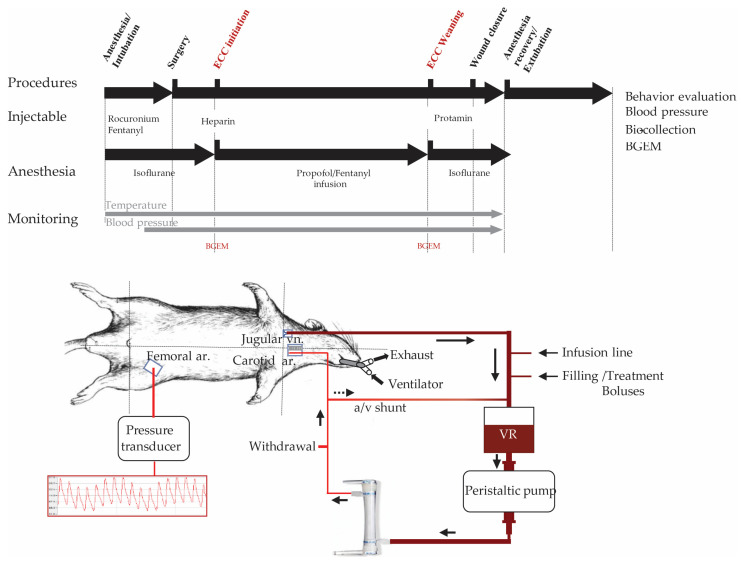
ECC montage, protocol, and hemodynamic monitoring a/v shunt: arterial/venous line shunt; VR: venous reservoir; ar.: femoral artery; vn.: vein. Protocol for ECC: Isoflurane was used for induction in a box at 5%, FiO_2_ 1.0, for maintenance with a mask at 2.5% and with an orotracheal probe (14 G), thanks to mechanical ventilation at 1.5%, FiO_2_ 1.0, 6 mL/kg tidal volume, 58 ± 2 cycle/min for maintenance of normocapnia. Fentanyl and rocuronium bromide boluses were administrated before intubation, respectively, at 12 µg/kg and 1 mg/kg, iv. During ECC, anesthesia was achieved with a propofol/ fentanyl infusion at, respectively, 30 mg/kg/h and 2 µg/kg/h. A total of 250 UI/kg unfractionated heparin was injected through the carotid catheter before initiation and was antagonized at the end of protocol, thanks to protamine sulfate. Extubating was achieved after the animal started breathing by itself. ECC: Extracorporeal circulation; BGEM: Blood gases electrolytes metabolites.

Animal preparation: The procedure (Figure 4) was established according to the regulatory asepsis conditions (NC3R, NIH guidelines). The animals were anaesthetized with 5% isoflurane/FiO_2_ 1.0 in an induction box and then maintained with a mask with 2% isoflurane. A rectal probe and a heating surgery platform (Harvard Apparatus, Molliston, MA, USA) were used to control and maintain the rats’ temperature at 37.5 °C ± 1 °C during surgery and 36.5 °C ± 1 °C during ECC. A bolus of fentanyl (12 µg/kg; Fentadon, Dechra, Montigny-le-bretonneux, France) was administered to ensure adequate pain relief during surgery and was slowly injected in the dorsal penile vein to avoid any respiratory failure. Prior to surgery, orotracheal intubation (14 G modified cannula; Instech laboratories, Plymouth, PA, USA) allowed anesthesia maintenance with 1.5% isoflurane/FiO_2_ 1.0. Mechanical ventilation was set (Harvard model 683, Harvard apparatus, Molliston, MA, USA) with a tidal volume of 6 mL/kg and a respiratory rate of 58 ± 2 cycles per minute. To achieve muscle relaxation during mechanical ventilation, the rats were curarized with an IV injection of rocuronium bromide (Esmeron, 1 mg/kg; Hospira, Paris, France).

Surgery: Three cannulas filled with heparinized Ringer-lactate (30 IU/mL) were respectively inserted (Figure 4) in the left femoral artery (PE-10; Instech laboratories, Plymouth, MA, USA) to monitor the systemic arterial blood pressure, in the right jugular vein (6Fr modified canula, Vygon, Écouen, France), and in the left carotid (PE-50; Instech laboratories, Plymouth, PA, USA). Unfractionated heparin (300 UI/kg; Panpharma, Beignon, France) was injected in the jugular vein before the initiation of ECC.

CPB initiation and maintenance: The arterial and venous cannulas were connected, respectively, to the 3-way valve next to the oxygenator and the venous reservoir. The pump was primed slowly and then the clamp on the venous line was removed. The pump speed was increased until the mean arterial pressure (MAP) minimum target of 60 mmHg was reached. Phenylephrine (15 µg; Renaudin, Itxassou, France) was only administered during the ECC period depending on the vitals. The criteria for starting phenylephrine infusion were a decrease in venous return with the venous canula rightly placed and a decrease in the mean arterial pressure without the possibility to further increase pump speed. Recovery of the MAP and venous return allowed us to decrease or stop the phenylephrine perfusion. The volume of phenylephrine was reported at the end of the ECC for each animal (Supplemental Figure S1: Sham: 00.0 ± 0 μg/h; ECC: 25.7 ± 2.7 μg/h, *p* < 0.0001).

The anesthesia was switched to total intravenous anesthesia (TIVA) with an infusion of propofol (30 mg/kg/h; PropoVet, Abott, Rungis, France) combined with fentanyl (2 µg/kg/h), thanks to a mechanical syringe pump (PHD 22/2000, Harvard Apparatus, Molliston, MA, USA). Extracorporeal circulation was maintained for 60 min.

Weaning and anesthesia recovery: The venous route was clamped and the blood contained in the tubing was restituted by the arterial cannula before stopping the pump. The sham rats were not affected by any volume restitution or vascular filling. The anesthesia was switched back to isoflurane 1.5%, FiO_2_ 100%. The carotid and femoral arteries and jugular vein were freed from their catheters and then terminally tied with sterile 5/0 non-resorbable threads (silk; B. Braun, France). Finally, all the wounds were closed with subcutaneous stitches (5/0 non-resorbable Vycril). The rats were maintained 10 to 15 min under anesthesia after weaning to fully recover and to warm up to 37 ± 0.5 °C if necessary. To initiate the awakening, isoflurane was gradually decreased from 1 to 0%. The rats were finally extubated at the onset of signs of resumption of spontaneous respiratory activity.

Post-operative care, monitoring, and biocollection: The rats weaned from anesthesia were placed in a heated chamber (30 °C) after receiving subcutaneous fluid therapy (10 mL/kg, NaCl 0.9%, B. Braun, France) mixed with buprenorphine (0.03 mg/kg, Buprecare^®^, Axience, Pantin, France). Five hours after extubating (H+5), the arterial pressure of the rats was measured, thanks to a catheter inserted in the right carotid artery under isoflurane anesthesia (1.5%, FiO_2_ 1.0). The rats were then euthanized, and the heart, kidneys, liver, brain, and blood were collected and snapped frozen in liquid nitrogen and then stored at −80 °C for further experimentations.

#### 4.2.3. Experimental Design and Groups

Hemodynamic and biochemical evaluations were made on 6 rats on ECC and on 6 rats with the sham procedure. The sham rats received every procedure and medication without connecting the cannulas to the ECC circuit. For the DGESeq study on PBMC, 15 rats were randomly submitted to sham or ECC procedure and were euthanized directly at the ECC end.

#### 4.2.4. Arterial Pressure, Temperature Monitoring, and Measure of Blood Samples

After sterile animal preparation, the arterial systemic blood pressure and heart rate were continuously recorded throughout the procedure with IOX software 2.9 (EMKA Technologies, Paris, France). After the orotracheal intubation, the blood pressure was recorded through the femoral artery catheter. The arterial blood was obtained before (ECC start) and after the ECC (ECC end) and at H+5 to measure the cell blood count/blood gases/electrolytes/metabolites (BGEM) on a point-of-care card reader (ePOC, BGEM card; Siemens Healthineers, Courbevoie, France) with hemodilution correction. The plasma was obtained from sampling at the ECC end and at H+5.

### 4.3. RNA Extraction, Quality Evaluation, and DGESeq Processing and Analyses

The RNA extraction from the lysed PBMC were processed according to the RNeasyPlus Mini Kit instruction manual and the RNA were recovered in 40 µL of sterile RNAse-free water (Qiagen, Les Ulis, France) and dosed with the NanoDrop ND-1000 Spectrophotometer (Thermo Scientific, Illkirch, France). Retro-transcription was achieved from 100 ng of RNA with the RT-superscript IV VILO kit. The sample preparation, verification of the sample quality and quantity, and transcriptomics were performed according to the protocol explained and detailed by Charpentier et al. [40]. The identification and selection of differentially expressed genes in humans was made with the “timecourse” package by a ranking method with a Hotelling T^2^ Score [41]. This method allowed the comparison of time-matched data and also allowed us to select the top 8% of the most differentially expressed genes in the largest number of patients. The package “clusteRprofiler” was used for enrichment analyses and associated graphical representation [42]. Gene ontology (GO), biological process, and KEGG (Kyoto encyclopedia of genes and genomes) enrichments to highlight the most statistically represented annotations of biological processes and signaling pathways among the selected genes GO and KEGG enrichment analyses were performed.

### 4.4. Statistical Analyses

The results were expressed as the mean ± SEM. Normality was tested thanks to the D’Agostino–Pearson normality test and equal variances have been tested for the determination of non-parametric tests use. The comparison of times and groups was performed using a paired two-way ANOVA Sidak’s multiple comparisons test or Friedman’s test. For one group, a comparison-over-time analysis was performed using Kruskal–Wallis or Friedman’s tests, and was then analyzed using a post-hoc Dunn’s test (GraphPad Prism 8). A *p* value < 0.05 was considered statistically significant. For clinical data and DEGseq secondary analyses in humans and rats, “questionR”, “arsenal”, “timecourse”, and “clusteRprofiler” packages were used with R (v4.1.1).

## 5. Conclusions

Overall, our data indicates that our rat model is highly comparable to humans and appears to be relevant both in the adaptation of the procedure, as well as in the response to extracorporeal circulation. The main difference is that the phenotype is more severe than that described in most patients, allowing this new model to develop additional organ dysfunctions and to potentially study them afterward.

## Figures and Tables

**Table 1:** Point-of-care measure of blood gases and electrolytes on arterial blood from the rat model.

		Start ECC (Mean ± SEM)	End ECC (Mean ± SEM)		H+5 (Mean ± SEM)	
pH	Sham	7.41 ± 0.02	7.37 ± 0.03		7.40 ± 0.02	
	ECC	7.42 ± 0.01	7.42 ± 0.04		7.41 ± 0.03	
pCO_2_ (mmHg)	Sham	45.47 ± 2.94	46.13 ± 1.88		43.28 ± 3.43	
	ECC	42.67 ± 2.52	39.93 ± 4.14		41.15 ± 3.50	
pO_2_ (mmHg)	Sham	287.17 ± 35.86	386.68 ± 43.61		329.4 ± 70.54	
	ECC	333.12 ± 48.52	436.42 ± 66.41		464.62 ± 43.87	
BE (mmol/L)	Sham	4.18 ± 0.59	2.37 ± 1.87		2.15 ± 2.11	
	ECC	3.15 ± 0.67	0.80 ± 1.13		1.17 ± 1.14	
HCO_3_^−^ (mmol/L)	Sham	28.72 ± 0.72	27.98 ± 1.17		27.59 ± 1.77	
	ECC	27.47 ± 0.79	25.4 ± 1.24		25.73 ± 1.08	
Ca^2+^ (mmol/L)	Sham	1.32 ± 0.02	1.35 ± 0.03		1.24 ± 0.03	
	ECC	1.29 ± 0.02	1.40 ± 0.02	*	1.10 ± 0.04	$$$***
Cl^-^ (mmol/L)	Sham	101.00 ± 0.73	102.33 ± 1.52		100.67 ± 1.56	
	ECC	101.33 ± 0.84	102.83 ± 1.17		104.17 ± 0.7	
K^+^ (mmol/L)	Sham	4.90 ± 0.11	5.07 ± 0.14		4.17 ± 0.15	
	ECC	4.62 ± 0.15	4.42 ± 0.11		5.04 ± 0.22	*
Na^+^ (mmol/L)	Sham	139.33 ± 0.56	141.33 ± 4.08		139.33 ± 0.95	
	ECC	138.73 ± 0.87	139.17 ± 0.79		140.17 ± 0.79	
AGAP	Sham	10.17 ± 0.79	10 ± 0.75		12.2 ± 1.45	
	ECC	10.50 ± 0.92	11.17 ± 1.17		10.77 ± 0.92	
AGAPK	Sham	14.83 ± 0.7	15.25 ± 0.84		16.2 ± 1.45	
	ECC	15.17 ± 0.95	15.5 ± 1.15		15.5 ± 0.99	

BE: Base excess, AGAP, and AGAPK: anion gap, ECC: cardiopulmonary bypass. Statistical differences were calculated with a Kruskall–Wallis test and a Dunn’s post-hoc test. * *p* < 0.05; *** *p* < 0.001 vs. ECC Start; $$$ *p* < 0.01 vs. sham.

**Table 2:** Description of demographics and clinical data from the cohort.

	**Overall (*n* = 41)**
**Sex (Male)**	36 (87.8%)
**Age (year)**	
Mean (SD)	66.1 (12.9)
Range	32.0–82.0
**Body mass index**	
Mean (SD)	26.0 (3.6)
Range	20.0–38.0
**Surgery Type**	
Mitral valve repair	5 (12.2%)
Mitral valve repair + tricuspid annuloplasty	7 (17.1%)
Mitral valve replacement + tricuspid annuloplasty	3 (7.3%)
Mitral valve replacement + CABG	1 (2.4%)
Aortic valve replacement	2 (4.9%)
Aortic valve replacement + tricuspid annuloplasty	3 (7.3%)
Aortic valve replacement + CABG	4 (9.8%)
CABG x3	5 (12.2)
CABG > 3	3 (7.3%)
Ascending aorta surgery (Bentall, Tirone David, etc.)	7 (17.1%)
Bentall + CABG	1 (2.4%)
**Redux**	2 (4.9%)
**Dyslipidemia**	25 (61.0%)
**Hypertension**	28 (68.3%)
**Tobacco**	
Smoker	4 (9.8%)
**Diabetes**	7 (17.1%)
**Obesity**	5 (12.2%)
**Familial history**	3 (7.3%)
**Exogenesis**	3 (7.3%)
**NYHA**	
0	4 (9.8%)
I	8 (19.5%)
II	24 (58.5%)
III	5 (12.2%)
**Arteriopathy**	8 (19.5%)
**Myocardial infarction**	11 (26.8%)
**Cerebral dysfunction**	1 (2.4%)
**Chronic renal failure**	9 (22.0%)
**Dialysis**	1 (2.4%)
**BPCO**	2 (4.9%)
**Asthma**	1 (2.4%)
**FeVG**	
Mean (SD)	60.8 (7.8)
Range	40.0–70.0
**EUROSCORE II**	
Mean (SD)	2.5 (2.2)
Range	0.8–11.6
**CPB time (min)**	
Mean (SD)	126.9 (37.5)
Range	63.0–229.0
**Cross-clamping time (min)**	
Mean (SD)	98.0 (30.2)
Range	45.0–182.0
**Catecholamine**	39 (95.1%)
**Norepinephrine**	37 (90.2%)
**Dobutamine**	20 (48.8%)
**Transfusion peri-op**	6 (14.6%)
**Cell saver**	41 (100.0%)
**VIS score 6 h post-op**	
Mean (SD)	7.6 (18.1)
Range	0.0–112.0
**KDIGO 24 h post-op**	
0	31 (75.6%)
1	7 (17.1%)
2	2 (4.9%)
3	1 (2.4%)
**Left ventricular dysfunction post-op**	17 (41.5%)
**Hydroxycortisone**	2 (4.9%)
**Ventilation duration (h)**	
Mean (SD)	13.0 (48.1)
Range	2.0–312.0
**Pulmonary infection**	1 (2.4%)
**Bleeding volume 6 h post-op (mL)**	
Mean (SD)	191.8 (116.5)
Range	70.0–500.0
**Transfusion in critical care**	4 (9.8%)
**Intensive care stay (day)**	
Mean (SD)	3.8 (5.9)
Range	1.0–34.0
**Hospital stay (day)**	
Mean (SD)	13.6 (6.1)
Range	6.0–34.0

Ao: Ascending aorta; CABG: coronary arterial bypass graft; NYHA: New York Heart Association classification; COPD: chronic obstructive pulmonary disease; LVEF: left ventricular ejection fraction; EUROSCORE: European System for Cardiac Operative Risk Evaluation; VIS Score: vasoactive-inotropic Score, KDIGO: kidney disease improving global outcomes.

## Data Availability

The data that support the findings of this study are available from the corresponding author upon reasonable request.

## Supplementary Materials

The following supporting information can be downloaded at: https://www.mdpi.com/article/10.3390/ijms24087338/s1.
